# Fipronil disturbs the antigen-specific immune responses and GABAergic gene expression in the ovalbumin-immunized BALB/c mice

**DOI:** 10.1186/s12917-024-03878-3

**Published:** 2024-01-22

**Authors:** Jui-Fang Kuo, Yin-Hua Cheng, Chun-Wei Tung, Chia-Chi Wang

**Affiliations:** 1 School of Veterinary Medicine, National Taiwan University, Taipei, Taiwan; 2 PhD Program in Toxicology, Kaohsiung Medical University, Kaohsiung, Taiwan; 3 Institute of Biotechnology and Pharmaceutical Research, National Health Research Institutes, Miaoli County, Taiwan

**Keywords:** Fipronil, Ovalbumin, Immunotoxicity, Antigen-specific immune responses, GABAergic signaling

## Abstract

**Background:**

Fipronil (FPN) is a broad-spectrum pesticide and commonly known as low toxicity to vertebrates. However, increasing evidence suggests that exposure to FPN might induce unexpected adverse effects in the liver, reproductive, and nervous systems. Until now, the influence of FPN on immune responses, especially T-cell responses has not been well examined. Our study is designed to investigate the immunotoxicity of FPN in ovalbumin (OVA)-sensitized mice. The mice were administered with FPN by oral gavage and immunized with OVA. Primary splenocytes were prepared to examine the viability and functionality of antigen-specific T cells ex vivo. The expression of T cell cytokines, upstream transcription factors, and GABAergic signaling genes was detected by qPCR.

**Results:**

Intragastric administration of FPN (1–10 mg/kg) for 11 doses did not show any significant clinical symptoms. The viability of antigen-stimulated splenocytes, the production of IL-2, IL-4, and IFN-γ by OVA-specific T cells, and the serum levels of OVA-specific IgG_1_ and IgG_2a_ were significantly increased in FPN-treated groups. The expression of the GABAergic signaling genes was notably altered by FPN. The GAD67 gene was significantly decreased, while the GABAR β2 and GABAR δ were increased.

**Conclusion:**

FPN disturbed antigen-specific immune responses by affecting GABAergic genes in vivo. We propose that the immunotoxic effects of FPN may enhance antigen-specific immunity by dysregulation of the negative regulation of GABAergic signaling on T cell immunity.

**Supplementary Information:**

The online version contains supplementary material available at 10.1186/s12917-024-03878-3.

## Background

Fipronil (FPN), an extensively used N-phenylpyrazole pesticide in agriculture and veterinary medicine, induces hyper-excitation neuronal toxicity by antagonizing insect γ-aminobutyric acid (GABA_A_)-gated chloride channels [1–5]. Although FPN is classified as a Class II moderately hazardous pesticide by the World Health Organization (WHO), FPN has been implicated in adverse health and environmental effects with non-target species toxicity [6]. As honeybees and dragonflies exhibit vulnerability to FPN, leading to its ban in the European Union in 2013 [7]. In addition, FPN has been associated with non-target organ toxicity including liver and kidney damage, thyroid dysfunction, and reproductive toxicity in non-target species [3, 8–10].

The increasing use of FPN has raised concerns about potential harm to human health from environmental exposure [11–13]. FPN residues have been found in 40% of U.S. households, and cases of acute illnesses have been reported associated with unintentional exposure, particularly through contact with pets treated with FPN-containing products [14, 15]. An increase in adverse reports of pets treated with FPN has led to increased scrutiny by the U.S. Environmental Protection Agency (EPA) on spot-on insecticides containing FPN. Although FPN is authorized for pest control of pets, it is prohibited in the EU for all food-producing animals. In 2017, FPN contamination was reported in eggs from 45 countries due to illegal use, highlighting the risk of exposure to FPN [13, 16–18]. These reports highlight the potential risks of FPN exposure in humans. Due to the potentially toxic effects of FPN on non-target species or non-target organs, further mechanistic toxicity studies are needed.

Essentially, the neuroendocrine and immune systems are considered to have bidirectional communication [19]. Unfortunately, compared to the well-documented neurotoxicity of FPN, fewer studies have focused on investigating the immunotoxic effects of FPN. Rats were orally administered 10% LD50 (9.7 mg/kg) of FPN for 30 days, resulting in histopathological alteration in the spleen and thymus tissue [20]. In addition, the serum levels of IL-4, IL-12, and IgE were slightly increased after exposure to FPN, suggesting that long-term exposure to FPN could increase allergic and inflammatory responses [20]. Exposure of mice from 4 weeks to 13 weeks of age with 0.5% LD50 FPN (~ 0.5 mg/kg) didn’t alter the spleen weight, however, the mitogenic proliferation of ConA or LPS-stimulated splenocytes was slightly decreased [21]. FPN directly decreased the production of IL-2 and IFN-γ in human lymphocytic Jurkat cells, suggesting that the T cells may be affected by FPN directly at non-cytotoxic concentrations [22]. As these data indicated the adverse effects of FPN on the immune system, however, little is known regarding the immunomodulatory effects of FPN on T helper 1 and T helper 2 immune balance in vivo. Furthermore, the underlying mechanism of immunotoxicity of FPN on T cell-dependent immune responses needs to be urgently clarified.

The critical inhibitory neurotransmitter GABA can be synthesized and released by the immune cells. Additionally, GABAergic signaling genes and functional proteins are expressed in mononuclear phagocytes and lymphocytes [23]. Numerous studies have elucidated the diverse roles of GABA in the immune system. GABA acts as an intercellular signaling molecule to modulate monocyte migration and to suppress T cell activation, proliferation, and cytokine production, through its receptor signaling [23–25]. GABA participates in T cell-mediated immunity *via* GABA transporters (GAT) and GABA receptors [26]. GABA treatment dose-dependently inhibited antigen-specific T cell proliferation and the T cell responses to foreign and self-antigens in vitro [27]. Moreover, the antigen-specific T-cell response could be directly inhibited by the GABAergic agents [28]. Considering the systemic insecticidal properties of FPN, known to antagonize GABA_A_ receptors in insects, the regulation of GABAergic genes in immune cells might be a potential mechanism of FPN-induced immunotoxicity. This study aimed to study the effects of FPN on antigen-specific T-cell immunity and T helper (Th) 1 and Th2 balance using the ovalbumin (OVA)-sensitized mouse model. The roles of GABAergic genes involved in FPN-induced immunotoxicity were further explored.

## Results

### Effects of FPN exposure on body weight, spleen index, and spleen cellularity in vivo

Mice exposed to corn oil (VH) or FPN (1, 5, 10 mg/kg) for a total of 11 doses did not exhibit any apparent clinical symptoms. Besides, no mortality was observed in FPN-treated groups. Administration of OVA-immunized mice with 5 and 10 mg/kg of FPN slightly slowed down weight gain and increased the spleen index compared to the VH group (Table 1). The cellularity of CD4^+^, CD8^+^, CD11b^+^, Gr1^+^, and B220^+^ in splenocytes was not altered during the administration of FPN (Table 1).

**Table 1:** Effects of FPN exposure on body weight, spleen index, and cellularity of splenocytes

	NA	VH	Fipronil (mg/kg)		
			1	5	10
**Body Weight**					
Day 1	21.39 ± 0.25	21.56 ± 017	21.24 ± 0.21	21.5 ± 0.21	21.68 ± 0.18
Day 16	23.68 ± 0.31	23.22 ± 0.24	22.46 ± 0.18	22.31 ± 0.23^*^	21.74 ± 0.19^*^
**Spleen index**	3.888 ± 0.07	4.124 ± 0.05	4.205 ± 0.1	4.211 ± 0.96	4.577 ± 0.26
**Spleen Cellularity (%)**					
CD4^+^	24.03 ± 0.43	21.9 ± 0.66	17.52 ± 1.63	18.22 ± 1.7	21.21 ± 0.51
CD8^+^	13.28 ± 0.61	14.23 ± 0.57	13.61 ± 0.7	13.67 ± 0.74	13.22 ± 0.78
B220^+^	50.94 ± 1.07	51.78 ± 1.4	52.75 ± 1.41	51.87 ± 1.13	49.76 ± 1.33
CD11b^+^	1.92 ± 0.34	1.49 ± 0.25	1.68 ± 0.3	1.5 ± 0.23	1.72 ± 0.32
Gr1^+^	1.18 ± 0.25	0.99 ± 0.19	1.03 ± 0.22	0.98 ± 0.2	1.03 ± 0.19
CD11b^+^/Gr1^+^	1.77 ± 0.14	2.29 ± 0.22	2.19 ± 0.21	2.34 ± 0.24	2.4 ± 0.24

^a^ Spleen index was calculated as the spleen weight (mg) per body weight (g). Data was expressed as mean ± SEM of 20 mice pooled from four independent experiments

^b^ Splenocytes were prepared as described in the Materials and Methods section. The percentage of CD4^+^, CD8^+^, B220^+^, CD11b^+^, and Gr1^+^ cells was determined by flow cytometry. Data was expressed as mean ± SEM pooled from four independent experiments (*n* = 20). *p < 0.05 as compared with the VH group

### Modulation of antigen-specific antibody production by FPN administration

**Fig. 1:**
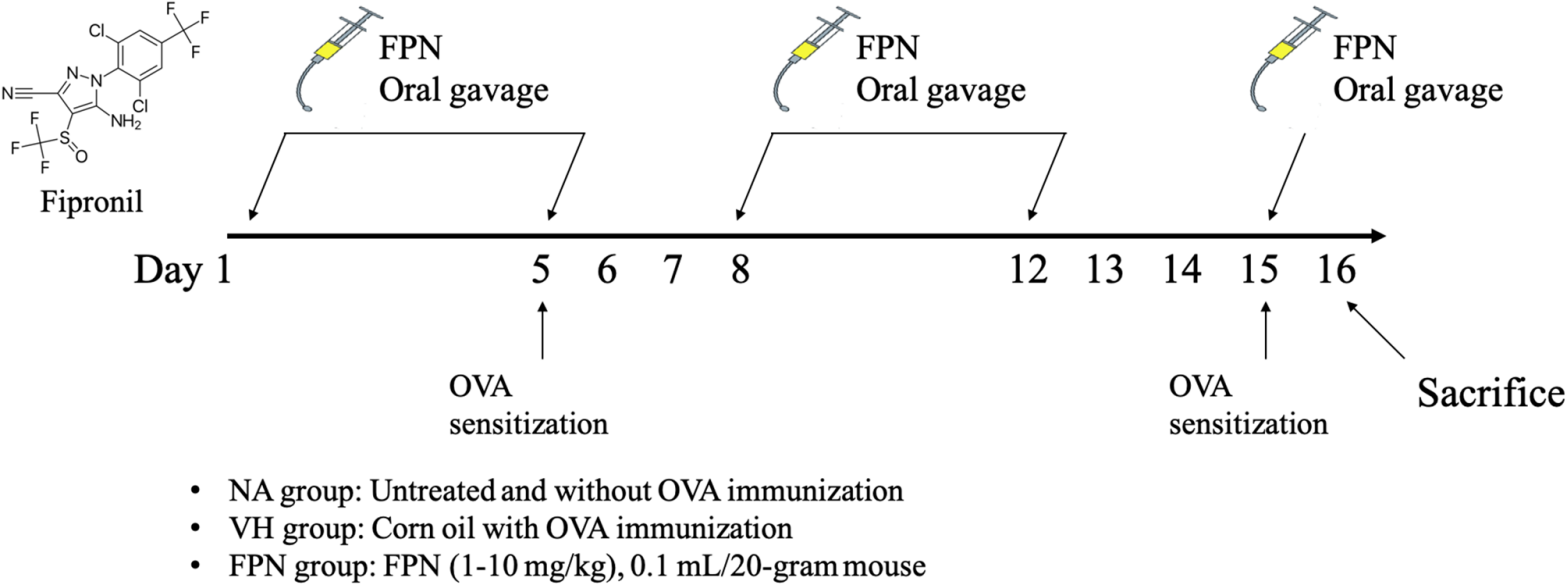
Protocol for fipronil (FPN) administration and ovalbumin (OVA) immunization. Mice were randomly divided into the following groups: naïve (NA), vehicle-treated and OVA-immunized (VH), and FPN-treated and OVA-immunized (FPN). The dosing regimen for FPN administration and antigen immunization was described in Materials and Methods

**Fig. 2:**
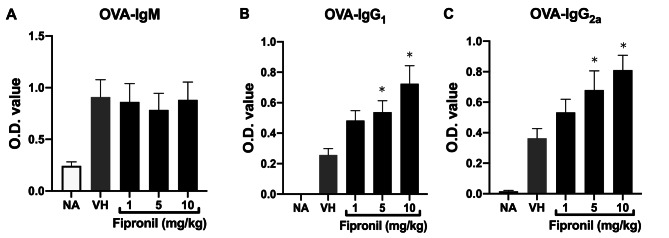
Induction of OVA-specific IgG_1_ and IgG_2a_ production in vivo. The serum levels of OVA-specific IgM, IgG_1_, and IgG_2a_ were determined by ELISA. Data was expressed as mean ± SEM of 20 individuals pooled from 4 independent experiments. *p < 0.05 as compared with the VH group

The BALB/c mice were immunized with OVA to study T-cell-dependent immune responses (Fig. 1). As expected, OVA immunization induced an appreciable increase in the serum levels of all three measured OVA-specific immunoglobulin (Ig), as compared to the non-immunized control (Fig. 2; NA vs. treatment groups). The serum value of OVA-specific IgG_1_ and IgG_2a_ was markedly increased in a dose-dependent manner (Fig. 2B-C) but the level of OVA-specific IgM was not altered by FPN (Fig. 2A).

### FPN enhanced the cell viability and disturbed IL-2, IL-4, and IFN-γ production ex vivo

**Fig. 3:**
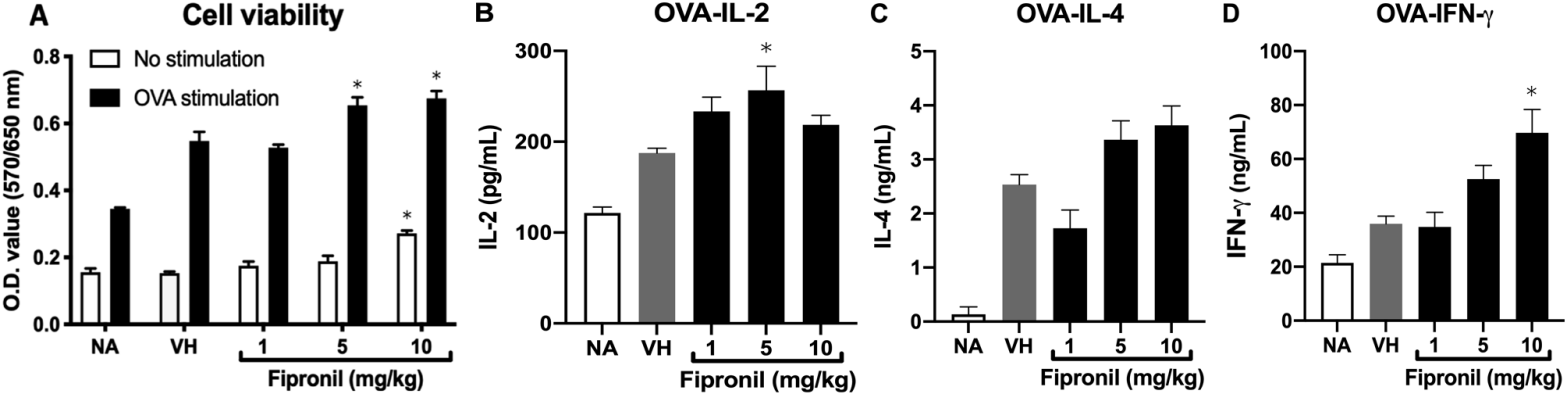
Enhancement of the cell viability and disturbance of IL-2, IL-4, and IFN-γ production. Splenocytes with the same cell concentration were prepared from each group of mice and cultured in the presence of ovalbumin (100 µg/mL) for 72 h. The supernatants were collected to measure the concentration of IL-2, IFN-γ, and IL-4 by ELISA. Data was expressed as the mean ± SEM of quadruplicate cultures and representative of four independent experiments (*n* = 20). *p < 0.05 was significant compared to the VH group

Next, we examined the effects of FPN on the proliferation of OVA-stimulated splenocytes using an MTT assay. FPN (5 and 10 mg/kg) robustly enhanced the cell viability of splenocytes in the absence or presence of OVA (100 µg/mL) (Fig. 3A). Naïve T cells proliferate and differentiate into effector Th cells, which are key effectors of the adaptive immune response, based on their secretion of cytokines. Th1 cells secrete IL-2 and IFN-γ, whereas Th2 cells secrete IL-4. The balance between Th1 and Th2 is required in an integrated immune system [29, 30]. Therefore, we investigate the effects of FPN on the production of Th cytokines. As shown in Fig. 3B-D, the production of IL-2, IL-4, and IFN-γ by splenocytes stimulated with OVA (100 µg/mL) was significantly increased at high-dose treatment groups (5, 10 mg/kg of FPN).

### FPN slightly down-regulated IL-2, IL-4, and GATA3 expression by OVA-stimulated splenocytes

Th1/Th2 cytokines play pivotal roles in modulating host immune responses [30]. As FPN increased antigen-specific cytokine production including IL-2, IL-4, and IFN-γ, we further examined the effects of FPN on the gene expression of Th1/Th2 cytokines and upstream transcription factors. Surprisingly, the relative mRNA expression of IL-2, IL-4, IFN-γ, GATA3, and T-bet by splenocytes stimulated with OVA (100 µg/mL) for 72 h were not altered (Fig. S1A-E).

### FPN altered GABAergic signaling gene expression by primary splenocytes

**Fig. 4:**
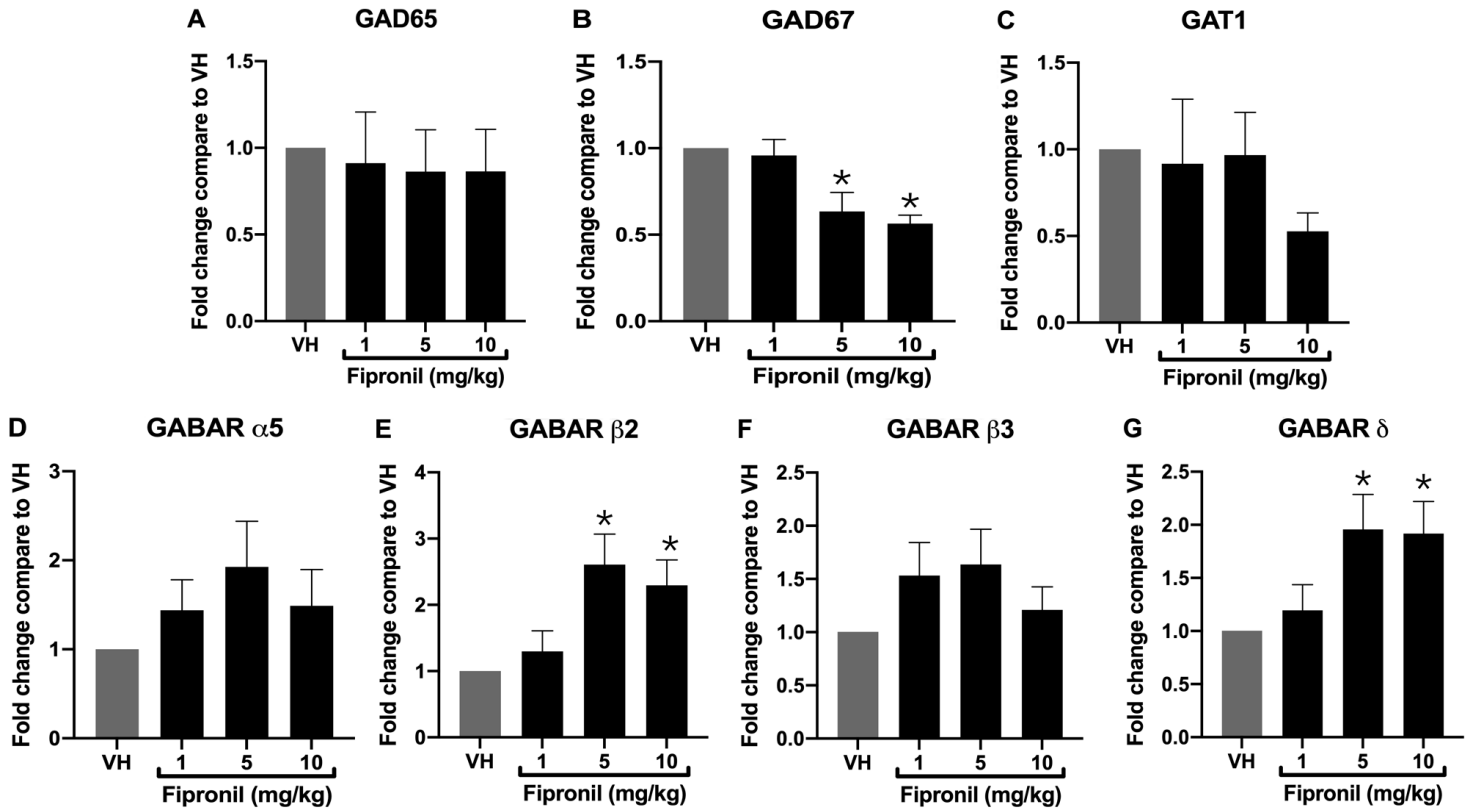
FPN significantly reduced the mRNA expression of GAD65 and GAT1 and altered the GABA receptor subunit expression. The total RNA of splenocytes (6 ⨉ 10^6^ cells) harvested from different treatment groups was extracted to detect the mRNA expression of GAD65, GAD67, GAT1, GABAR α5, GABAR β2, GABAR β3, and GABAR δ by qPCR. The expression level of HPRT was used as the control for semi-quantification. Results were expressed as the mean ± SEM pooled from four independent experiments with technological duplication in each group (*n* = 20). *p < 0.05 was significant compared to the VH group

FPN is a systemic insecticide known to antagonize the GABA_A_ receptors in insects. To investigate whether the GABAergic signaling genes are involved in the disturbances of immune responses by FPN exposure, the expression of glutamate decarboxylases (GAD65 and GAD67), GABA transporter gene (GAT1), and GABA receptor subunit (GABAR α5, β2, β3, δ) were further examined in splenocytes isolated from FPN-treated mice. The results showed that FPN notably reduced the expression of GAD67 genes in a dose-dependent manner, and then the expression of GABAR β2 and GABAR δ genes were significantly increased at the high dose of FPN compared to VH control. The levels of GAT1 mRNA are also decreased at 10 mg/kg FPN but no remarkable difference (Fig. 4A-G).

## Discussion

The present results demonstrated that exposure to FPN disturbed antigen-specific immune responses in vivo. Compared with extensive studies of FPN-induced neurotoxic, reproductive, and cytotoxic effects [9, 31–33], limited studies have explored immunotoxicity associated with lymphocyte functionality [20]. Herein, we focused on mature T cells that primarily reflect T-cell-dependent immune responses. To the best of our knowledge, this study is the first report on the immunotoxic effects of FPN on antigen-specific immunity by oral exposure. Once FPN was administrated to mice by oral gavage, it rapidly transformed into a more toxic metabolite [34, 35], fipronil sulfone, and accumulated for an extended period in the plasma [36]. Furthermore, fipronil sulfone remained at a stable and consistent level in the plasma after 4 times oral administration of FPN [36]. During the same dosing regimen of FPN administration by oral gavage (5 days/week, up to 3 weeks), the plasma level of fipronil sulfone slightly increased. This data indicated the stable bioaccumulation profile of FPN metabolites in mice through oral administration [36]. We speculate that FPN metabolites can stably accumulate in the body to affect the immune responses. However, the intermittent recovery period could have resulted in an underestimation of the effects of the lower dosage of FPN on immune responses. A long-term exposure to FPN needs to be studied to evaluate the prolonged effects of FPN on T-cell immune responses.

In this study, exposure to FPN (1–10 mg/kg) for 11 doses did not induce severe clinical symptoms, and there were no significant changes in spleen index and spleen cellularity. Similar to the findings of Farhad and Banalata (2020), no mortality was recorded [21]. Notably, the body weight gain in the FPN (5 and 10 mg/kg) groups on day 16 exhibited a slight decrease, while the spleen index marginally increased in the 10 mg/kg group. The moderate decline in body weight may be attributed to oxidative stress induced by FPN (the dosage used was ≤ 9.7 mg/kg (1/10 LD50)) [37]. Nevertheless, these observations suggest that administering 10 mg/kg of FPN to mice could potentially induce mild toxicity. Combined with the spleen cellularity data (both populations of lymphocytes and myeloid cells were not significantly altered), a slight increase in the spleen index in the 10 mg/kg group may be due to decreased body weight. Another report showed that exposure of rats to 9.7 mg/kg of FPN for 30 days caused histopathological changes in the spleen. A significant reduction in the proportion of white pulp area was found, accompanied by severe atrophy of lymphoid follicles [20]. Although no changes in splenic composition were observed in our current data, the effects of FPN on lymphopoiesis may become more pronounced with increasing exposure time.

Dysregulated Th1/Th2 cytokine production has the potential to contribute to the development of autoimmune disorders and allergic conditions [29, 30]. In the present study, serum levels of OVA-specific IgG_1_ and IgG_2a_ were markedly increased in FPN-treated mice, suggesting that FPN interfered with T cell-dependent antibody production. Furthermore, FPN administration at high dose enhanced the viability and proliferation of antigen-specific T cells in response to OVA stimulation ex vivo, which subsequently led to a significant increase in the production of IL-2, IL-4, and IFN-γ cytokines by OVA-specific T cells. These results revealed the immunostimulatory effects of FPN on adaptive immune responses. The immunostimulatory effects of FPN have been reported in human nasal epithelial cells. Both protein levels and mRNA expression of pro-inflammatory cytokines including IL-1 beta, IL-6, and IL-8 were induced by FPN in vitro through activation of ERK1/2 MAPK, p38 MAPK, and the NF-κB pathway [38]. On the contrary, the mRNA expression of Th1/Th2 cytokines and their respective upstream transcription factors remained unchanged or slightly decreased in the present work. Since the immunostimulatory effects of FPN on Th1/Th2 cytokines are not correlated with the mRNA expression of Th1/Th2 cytokines and differentiation genes, it is suggested that the immunomodulatory effects of FPN on antigen-specific T cell responses may not be through the regulation of Th1/Th2 differentiation and cytokine gene expression.

As FPN is antagonistic to GABA receptors, the regulation of the GABAergic genes by FPN was further investigated to clarify their roles in FPN-mediated immunomodulatory effects in vivo. GABA inhibited antigen-specific T cell proliferation and the T cell responses to foreign and self-antigens in a dose-dependent manner in vitro [27]. The antigen-specific T-cell immune responses were inhibited by the GABAergic agents. The levels of inflammatory cytokines are reduced after GABA treatment in peripheral macrophages [28]. GABA and/or GABA_A_ receptor agonists reduced inflammatory responses, antigen-specific cytotoxic immune responses, and antigen-primed delayed-type hypersensitivity reactions, in a non-obese diabetic mouse model of type 1 diabetes [27, 28, 39]. Collectively, these lines of evidence indicate the regulatory roles of GABAergic signaling in the over-reactive adaptive immune responses.

Glutamate decarboxylase (GAD) is an enzyme that catalyzes the decarboxylation of glutamate to GABA. GABA secretion has been observed in stimulated T cells when cultured in a conditioned medium. In addition, dendritic cells and macrophages express GAD65 transcripts to synthesize GABA. Although the main GAD in stimulated T cells has not been well studied, the secretion of the bulk GABA by T cells may be impacted by re-uptaking, storage, and secretion of GABA [28]. In the present study, the expression of GAD67, an enzyme that could be expressed in B cells [40], by antigen-stimulated splenocytes was significantly reduced in FPN-treated mice in a dose-dependent manner. This data indicated that FPN might interfere with the synthesis of GABA through the down-regulation of GAD67 in splenocytes. GABA transporter type 1 (GAT1) is primarily engaged in GABA binding and transport from the cytoplasm to the extracellular space (reverse mode) and back into the cytoplasm (forward mode). Dysfunctional GAT1 may lead to a delay in communication with post-synaptic GABA receptors, resulting in a variety of neurological diseases [41]. The CD4^+^ T cells isolated from GAT1^−/−^ mice have higher IL-2 and IFN-γ secretion under conditional stimulation, promoting T cell activation and survival through PKC-dependent signaling pathways [42]. These results of previous literature are also consistent with our cytokine data, where a significant increase in the secretion of IL-2 and IFN-γ was observed in the FPN-treated groups. Furthermore, a slight decrease in the expression of GAT1 mRNA has been shown, suggesting the impairment of GABA recycling may be involved in the immunostimulatory effects of FPN.

**Fig. 5:**
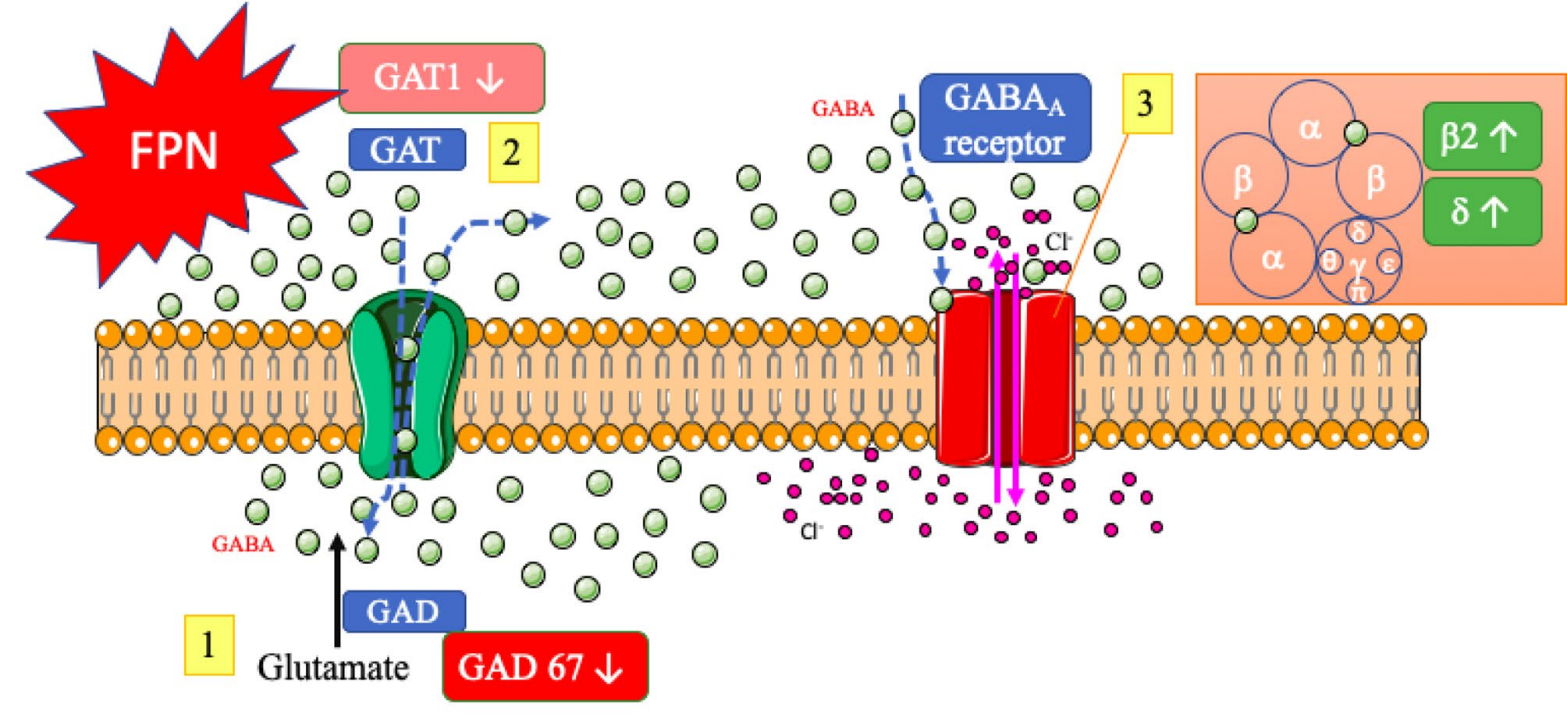
A schematic diagram of the synthesis and transport of GABA signaling affected by FPN in this study. (1) GABA is produced enzymatically through the activity of glutamate decarboxylase (GAD 65/67). The FPN exposure significantly decreased the mRNA levels of GAD 67, which might reduce GABA synthesis. (2) GABA transporters (GAT) facilitate the transportation of GABA through the cellular space. The deficiency of GAT1 would affect the function of GABA transportation and might be associated with IL-2 and IFN-γ increase by FPN exposure. (3) Activation of GABA_A_ receptors occurs upon GABA binding, leading to either the efflux or influx of Cl^−^. After FPN treatment, the mRNA expression of subunits of β2 and δ were notably increased which might be a compensatory mechanism to overcome the adverse effects of FPN-induced dysregulation of GABAergic genes. Parts of the figure were drawn by using pictures from Servier Medical Art. Servier Medical Art by Servier is licensed under a Creative Commons Attribution 3.0 Unported License (https://creativecommons.org/licenses/by/3.0/)

Emma L. Sparrow et al. identify that the GABA_A_ receptors are expressed in human and mouse immune cells, and the main subunits of mouse T cells are α5, β2, β3, and δ [43]. Although T cells can form different heteropentameric GABA_A_ receptor subtypes, the previous study hasn’t completely demonstrated the composition and physiological relevance of the GABA_A_ receptor subunit on T cells, because their affinity for GABA and pharmacological properties are varied in different subunits of the GABA_A_ receptor. Nevertheless, through functional GABA_A_ receptors but not GABA_B_ receptors, higher levels of GABA could reduce antigen-primed T cell proliferation to inhibit immune responses [27]. Our present study demonstrated that the expression of GABA receptor genes was altered after exposure to FPN. The mRNA expression of subunits of β2 and δ were notably increased, and the α5 and β3 transcripts were slightly induced at 1 and 5 mg/kg groups. As FPN competitively binds to GABA receptors, resulting in a reduction of GABA’s regulatory effects on immune responses, we speculated that the up-regulation of GABA receptor genes may be a compensatory mechanism to overcome the adverse effects of FPN-induced dysregulation of GABAergic genes. In our data without the obvious changes in spleen composition, the alterations of GABAergic genes were found in concordance with the FPN-mediated abnormal immunostimulatory effects, suggesting the dysregulation of GABAergic genes may play a role in the immunotoxicity of FPN. Figure 5 summarizes the effects of FPN on GABAergic genes in this study.

In conclusion, our study showed that oral exposure to FPN for 11 administration schedule disrupted antigen-specific T-cell responses in vivo, demonstrating immunotoxic effects with intermittent exposure. As GABAergic components regulate T cell-mediated immunity, we hypothesized that FPN may augment the antigen-specific immunity *via* dysregulation of GABAergic genes. This study may open an avenue to investigate the immunotoxic effects of FPN on the dysregulation of GABAergic signaling in primary immune cells.

## Materials and methods

### Reagents

All reagents were purchased from Sigma (MO, USA) unless otherwise stated. Fipronil (FPN, purity ≧ 97%) was purchased from Tokyo Chemical Industry Co., Ltd. (Tokyo, Japan) and dissolved in corn oil. RPMI 1640 medium (Cat. No. SH30027.02) was purchased from Hyclone (UT, USA). Fetal bovine serum (FBS, Cat. No. 10437-028) and cell culture reagents were purchased from GIBCO BRL (MD, USA) and GE Healthcare (Chicago, IL). Reagents used for ELISA examination were purchased from BD Biosciences (San Jose, CA).

### Experimental animals

All animal experiments were approved by the Institutional Animal Care and Use Committee of the National Taiwan University (IACUC Approval No: NTU108-EL-00026). Male BALB/c mice aged 5 weeks (weight 18–20 g) were purchased from BioLASCO Experimental Animal Center (Taiwan Co., Ltd, BioLASCO, Taipei, Taiwan). Randomization was carried out as follows: upon arrival, each mouse was assigned to a group and weighed. The total number of mice was then weighed and divided into five groups based on their weight to minimize initial weight differences within each group. Subsequently, the mice were randomly transferred into plastic cages containing sawdust bedding and quarantined for one week (3 − 6 mice per cage). Mice were raised in temperature (24 ± 2 °C) and humidity (50 ± 20%) controlled room on a 12 h light/dark cycle and given standard laboratory food and water *ad libitum*.

### Protocol of animal experiments

The mice (5 animals/group) were either left untreated (naïve; NA) or administered by oral gavage with FPN (1, 5, 10 mg/kg) suspended in corn oil and/ or vehicle (VH; corn oil) 5 days per week for 2 weeks and one more dose before second OVA sensitization on day 15 (Fig. 1). Based on general immunotoxicity assessment, the U.S. Food & Drug Administration recommends a common and ideal functional assay for detecting potential immunotoxicity of drugs, T-cell dependent antigen response (TDAR) assay, triggering by exogenous protein as antigen like ovalbumin (OVA) to induce T-cell–dependent immune responses [44]. This assay assesses immunological function, which is determined by the efficiency of several immune processes, such as antigen uptake and presentation, T cell help, B cell activation, and antibody generation [45, 46]. Except for the NA group, mice were sensitized with OVA twice by intraperitoneal injection, with 0.1 mL sensitization solution containing 100 µg ovalbumin and 1 mg alum (as adjuvant) in saline on day 5 and day 15. Mice were sacrificed on day 16, and their serum samples and spleens were harvested for further experimentation. Because the mice had to be observed for clinical changes after administration of FPN, the experimenter could not be blinded to whether the animal was exposed to FPN or corn oil.

### Splenocyte isolation and culture

Mice were sacrificed by cervical dislocation. The spleen was aseptically removed, washed, and extracted into single-cell suspensions, and the erythrocytes in splenocyte cultures were lysed by ACK buffer (0.15 M NH_4_Cl, 0.01 M KHCO_3_, 0.1 mM Na_2_EDTA, pH 7.4). The cells were cultured in RPMI 1640 medium supplemented with 5% heat-inactivated FBS, 100 U/mL penicillin, and 100 µg/mL streptomycin, and cultured at 37 °C in 5% CO_2_ for further experiments.

### Spleen index

The spleen of each mouse (*n* = 20 in each group) was dissected and weighed immediately after sacrifice. The spleen index was calculated as the spleen weight (mg) per body weight (g).

### Flow cytometric analysis for cellularity of splenocytes

The expression of CD4^+^, CD8^+^, CD11b^+^, Gr-1^+^, and B220^+^ by splenocytes was measured by flow cytometry. Briefly, splenocytes were stained with rat anti-mouse CD4 (BD Biosciences, San Jose, CA) and Gr-1 (eBioscience, Waltham, MA) conjugated with FITC and/or rat anti-mouse CD8 (BD Biosciences, San Jose, CA) and B220 (BD Biosciences, San Jose, CA) conjugated with PE-Cy5 and/or rat anti-mouse CD11b (eBioscience, Waltham, MA) conjugated with APC antibodies in staining buffer (PBS containing 2% FBS and 0.09% sodium azide) avoiding light on ice for 30 min. Appropriate rat anti-mouse antibodies were applied as the isotype control for evaluating non-specific binding. After washing, the single-cell fluorescence of 10,000 cells for each sample was measured by a flow cytometer (BD FACSCalibur, San Jose, CA) Data was analyzed by Flowjo 10.4 software (FlowJo LLC, Ashland, OR).

### Metabolic activity assay of splenocyte

The metabolic activity was determined by the 3-(4,5-dimethylthiazol-2-yl)-2,5-diphenyl-tetrazolium bromide (MTT) assay [47]. Splenocytes (6 × 10^6^ cells/mL) were cultured in 96-well plates with the presence of OVA (100 µg/mL) for 72 h. After OVA stimulation for 68 h, an MTT stock solution (5 mg/mL) was added and incubated for 4 h. Then, the formed formazan was dissolved by adding 100 µL Dimethyl sulfoxide (DMSO). The plate was read using an ELISA microplate reader (SpectraMax ® M5 Microplate Reader, Molecular Devices LLC, San Jose, California, USA) at OD_570 nm_ using OD_630 nm_ as a background reference.

### Measurement of cytokines and OVA-specific ig expression by enzyme-linked immunosorbent assay (ELISA)

Splenocytes (6 ⨉ 10^6^ cells/mL) were cultured in quadruplication in 48-well culture plates (0.3 mL/well). The levels of IL-2, IL-4, and IFN-γ in the culture supernatant with/without OVA stimulation for 72 h, OVA-IgM, IgG_1_, and IgG_2a_ in serum samples were determined by ELISA as previously described. The optical density was measured at OD_450 nm_ using an ELISA microplate reader (SpectraMax ® M5 Microplate Reader, Molecular Devices LLC, San Jose, California, USA).

### RNA isolation and quantitative polymerase chain reaction (qPCR)

The splenocytes incubated with OVA for 72 h were collected and homogenized in TRIzol reagent. The total RNA was isolated by the GENEzol Pure Kit (Geneaid Biotech Ltd., New Taipei City, Taiwan) according to the manufacturer’s instructions. The RNA concentration was quantified and qualified using the determination of OD_260 nm_, OD_280 nm_, and OD_230 nm_ by Nanophotometer™ (Implen GmbH, Munich, Germany). One mg of total RNA was reverse-transcribed by SensiFAST cDNA Synthesis Kit (BIOLINE, Memphis, TN) into cDNA products. Real-time PCR was performed by AriaMx Real-Time PCR System (Aglient Technologies, Santa Clara, CA). During the real-time PCR process, SensiFAST SYBR Lo-ROX Kit was provided to quantify mRNA expression. The expression of the HPRT gene was employed as an endogenous control to normalize the expression of target genes. The primers of the target gene used in this study are listed in Table 2.

**Table 2:** List of quantitative PCR primers

Gene name	Primers (5’ to 3’)
IL-2	F: AGCAGCTGTTGATGGACCTA R: CGCAGAGGTCCAAGTTCAT
IL-4	F: GCTAGTTGTCATCCTGCTCTTC R: GGCGTCCCTTCTCCTGTG
IFN-γ	F: GCCAAGTTTGAGGTCAACAAC R: CCGAATCAGCAGCGACTC
T-bet	F: GCCAGGGAACCGCTTATATG R: GACGATCATCTGGGTCACATTCT
GATA3	F: TACCCTCCGGCTTCATCCT R: TGCACCTGATACTTGAGGCAC
GAD65	F: TCAACTAAGTCCCACCCTAAG R: CCCTGTAGAGTCAATACCTGC
GAD67	F: CGCTTGGCTTTGGAACCGACAA R: GAATGCTCCGTAAACAGTCGTGC
GAT1	F: CAAGCCCAAAACCCTGGTAGT R: CCACGCAGGACATGAGGAA
GABAR α5	F: GATTGTGTTCCCCATCTTGTTTGGC R: TTACTTTGGAGAGGTGGCCCCTTTT
GABAR β2	F: GCTGGTGAGGAAATCTCGGTCCC R: CATGCGCACGGCGTACCAAA
GABAR β3	F: GAGCGTAAACGACCCCGGGAA R: GGGACCCCCGAAGTCGGGTCT
GABAR δ	F: TCAAATCGGCTGGCCAGTTCCC R: GCACGGCTGCCTGGCTAATCC
HPRT	F: TCAGTCAACGGGGGACATAAA R: GGGGCTGTACTGCTTAACCAG

### Statistical analysis

All the data were analyzed by SigmaPlot 14.0 (San Jose, CA). The mean ± standard error (SEM) was determined for each treatment group in the individual experiments. All analysis was executed in a blinded manner. Statistical differences between groups were evaluated by one-way ANOVA and Dunnett’s two-tailed t-test was employed to compare FPN-treated groups to the control group. *P*-value < 0.05 was defined as a statistical significance.

### Electronic supplementary material

Below is the link to the electronic supplementary material.

Supplementary Material 1

## Data Availability

The raw data used and/or analyzed during the current study are available from the corresponding author upon reasonable request.

## Abbreviations

FPN: Fipronil
OVA: Ovalbumin
GABA_A_: γ-aminobutyric acid
GAD: glutamate decarboxylases
GAT: GABA transporter
GABAR: GABA receptor
