# Supplementary material for: Fipronil disturbs the antigen-specific immune responses and GABAergic gene expression in the ovalbumin-immunized BALB/c mice
Source: BMC Vet Res. 2024 Jan 22;20:30. doi: 10.1186/s12917-024-03878-3 (PMC10801957; doi:10.1186/s12917-024-03878-3)
Supplement: Supplementary file 1 — Supplementary Material 1 [file 12917_2024_3878_MOESM1_ESM.pdf]

Supplementary Figures and Legends

Fig. S1. IL-2, IL-4, and GATA3 mRNA expression by OVA-stimulated splenocytes.

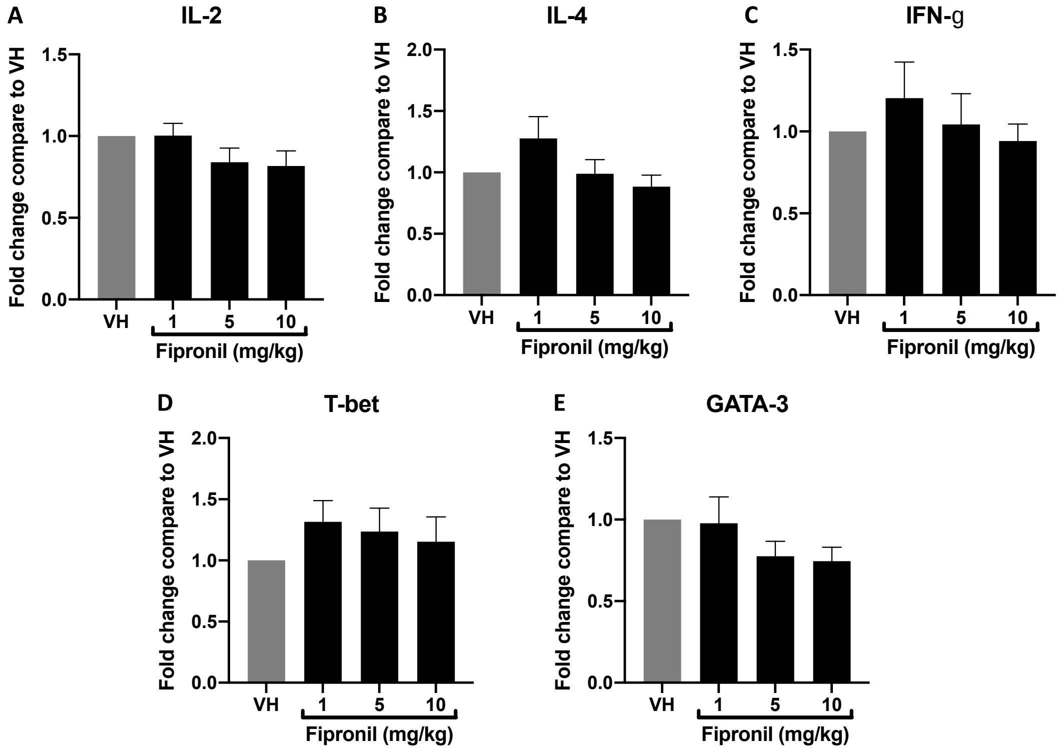

The total RNA of splenocytes (6x10^6 cells) harvested from different treatment groups was extracted to detect the mRNA expression of IL-2, IL-4, IFN-γ, T-bet, and GATA3 by qPCR after cultured with the presence of ovalbumin (100 μg/mL) for 72 h. The expression level of HPRT was used as the control for semi-quantification. Results were expressed as the mean ± SEM of pooled data from duplicate pooled from four independent experiments. *p<0.05 was significant compared to the VH group.
